# Short-Term Effects of Carbon Monoxide on Mortality: An Analysis within the APHEA Project

**DOI:** 10.1289/ehp.10375

**Published:** 2007-08-16

**Authors:** Evangelia Samoli, Giota Touloumi, Joel Schwartz, Hugh Ross Anderson, Christian Schindler, Bertil Forsberg, Maria Angela Vigotti, Judith Vonk, Mitja Košnik, Jiri Skorkovsky, Klea Katsouyanni

**Affiliations:** 1 Department of Hygiene and Epidemiology, University of Athens Medical School, Athens, Greece; 2 Harvard School of Public Health, Boston, Massachusetts, USA; 3 Community Health Sciences, St. George’s, University of London, London, United Kingdom; 4 Institute of Social and Preventive Medicine, University of Basel, Basel, Switzerland; 5 Department of Public Health and Clinical Medicine, Umea University, Umea, Sweden; 6 Universita degli studi di Pisa, Pisa, Italy; 7 Department of Epidemiology and Statistics, University of Groningen, Groningen, the Netherlands; 8 Institute of Public Health, Department of Environmental Health, Ljubljana, Slovenia; 9 Institute of Hygiene, Teplice, Czech Republic

**Keywords:** air pollution, carbon monoxide, heterogeneity, modeling, mortality

## Abstract

**Objectives:**

We investigated the short-term effects of carbon monoxide on total and cardiovascular mortality in 19 European cities participating in the APHEA-2 (Air Pollution and Health: A European Approach) project.

**Methods:**

We examined the association using hierarchical models implemented in two stages. In the first stage, data from each city were analyzed separately, whereas in the second stage the city-specific air pollution estimates were regressed on city-specific covariates to obtain overall estimates and to explore sources of possible heterogeneity. We evaluated the sensitivity of our results by applying different degrees of smoothing for seasonality control in the city-specific analysis.

**Results:**

We found significant associations of CO with total and cardiovascular mortality. A 1-mg/m^3^ increase in the 2-day mean of CO levels was associated with a 1.20% [95% confidence interval (CI), 0.63–1.77%] increase in total deaths and a 1.25% (95% CI, 0.30–2.21%) increase in cardiovascular deaths. There was indication of confounding with black smoke and nitrogen dioxide, but the pollutant-adjusted effect of CO on mortality remained at least marginally statistically significant. The effect of CO on total and cardiovascular mortality was observed mainly in western and southern European cities and was larger when the standardized mortality rate was lower.

**Conclusions:**

The results of this large study are consistent with an independent effect of CO on mortality. The heterogeneity found in the effect estimates among cities may be explained partly by specific city characteristics.

Adverse short-term effects of air pollution on health have been documented in recent years (Katsouyanni et al. 2001; Pope et al. 1995; Samet et al. 2000; Schwartz and Dockery 1992; Touloumi et al. 1996). The pollution indicators were mainly ambient particles (Katsouyanni et al. 1997; Pope and Dockery 1999; Samet et al. 2000), but gaseous pollutants such as nitrogen dioxide, ozone, and carbon monoxide have also been associated with adverse effects on mortality and morbidity (Samoli et al. 2006; Schwartz 1997; Touloumi et al. 1996). The results from epidemiologic studies have led to revisions of air quality guidelines and standards and scheduled dates for regular revisions in the future [European Commission (EC) 1999; U.S. Environmental Protection Agency (EPA) 1996; World Health Organization (WHO) 2000].

CO is produced by incomplete combustion of hydrocarbons. Its main source is vehicle exhaust emissions; secondary important sources are industry, heating, and fires. The concentrations of CO as well as their fluctuations are related to a large extent to the circulation of cars. The introduction of catalytic converters led to substantial reductions in ambient CO levels (Viras et al. 1996). CO concentrations are spatially heterogeneous within a city, with higher concentrations on busy roads and especially in street canyons. In addition, CO concentrations in cars and buses are much higher than ambient levels, and can account for an important part of exposure. Because of the spatial variability, levels of CO measured at fixed monitoring stations depend more on site characteristics than do levels of other pollutants. Indoor air can also be contaminated with high levels of CO, because space heaters fueled with oil, gas, or kerosene, gas stoves, and tobacco smoking cause significant emissions of CO. However, Georgoulis et al. (2002) have shown that ambient CO levels are a significant correlate and determinant of CO 48-hr personal exposure, and in fact the only consistent one between the five European cities included in their analysis.

CO is one of the few ambient air pollutants for which we know its biologically toxic form, carboxyhemoglobin (COHb). Binding of CO in the lungs with hemoglobin in the blood forms COHb, which reduces the oxygen-carrying capacity of the blood and impairs the release of oxygen from hemoglobin to extravascular tissues. These are the main causes of tissue hypoxia produced by CO at low exposure levels. The toxic effects of CO become evident in organs and tissues with high oxygen consumption such as the brain, the heart, exercising skeletal muscle, and the developing fetus. Severe hypoxia due to acute CO poisoning may cause both reversible, short-lasting neurologic deficits and severe, often delayed neurologic damage (WHO 2000). The mechanisms for effects of low-level exposure are unclear, but likely include reduced exercise capacity and exacerbation of cardiovascular symptoms in persons with coronary heart or lung disease. Reduced oxygen-carrying capacity of hemoglobin predisposes toward cardiac ischemia in persons with coronary artery disease. CO and traffic related co-pollutants have also been associated with alteration of the cardiac autonomic regulation in population-based studies (Liao et al. 2004) and in panel studies (Dales 2004; Riojas-Rodriges et al. 2006; Timonen et al. 2006). Ambient daily CO levels have been associated with increases in daily mortality and hospital admissions for cardiovascular diseases. The latter persists even at very low CO levels, suggesting that there is no threshold for the onset of these effects. Whether the relation between daily mortality and exposure to CO is causal or whether CO might act as a proxy for combustion particles is still an open question. Ambient CO may act through other pathways than COHb formation, and at lower levels those pathways may dominate the adverse health effects (Schwela 2000).

One key limitation of previous studies has been the use of data from a single or a few locations. We address this limitation by presenting the results of analyses investigating the short term effects of CO on mortality from all and cardiovascular causes within the APHEA-2 project (Air Pollution and Health: A European Approach 2; Katsouyanni et al. 1996), which uses an extensive European database of cities spanning across the Continent. This database also allows a comprehensive and structured approach at the second stage of the analysis, in which we explore the role of effect modifiers in explaining the heterogeneity of the relation of air pollution and mortality across cities.

## Materials and Methods

### Data

The APHEA-2 project is a multicenter study including 30 cities across Europe and associated regions that studied short-term health effects of air pollution. Data were collected on daily counts of all-cause mortality, excluding deaths from external causes [*International Classification of Diseases, 9th Revision* (ICD-9; WHO 1975) codes > 800] and cardiovascular (CVD) mortality (ICD-9 codes 390–459). The data covered at least 3 consecutive years for each city within the years 1990–1997. Details about the data have been published elsewhere (Katsouyanni et al. 2001).

Daily air pollution measurements were provided by the monitoring networks established in each town participating in the APHEA-2 project (Katsouyanni et al. 1996). Nineteen cities provided data on CO. A clear seasonal pattern was evident in all cities, with higher levels during winter. The mean correlation between the CO measurements in the different monitoring sites used ranged from 0.45 in Athens to 0.88 in Zurich. Time-series data on daily temperature (°C, daily mean) were used to control for the potential confounding effects of weather.

There is evidence that the average of 2 days’ pollution correlates better with mortality than a single day’s exposure (Schwartz 2000), and therefore we made an *a priori* decision to study the average of lags 0 and 1 for CO.

Table 1 presents descriptive characteristics of the individual city data sets. Together, they comprise a population of more than 40 million people. The Netherlands is considered one urban area because of its relative small size and dense population. The mean daily total number of deaths ranged from 6 to 342. For cardiovascular mortality, daily number of deaths ranged from 6 to 143. The mean levels of CO (maximum 8-hr average) ranged from 0.6 to 6.1 mg/m^3^. In the various cities, the correlation between CO and particulate matter < 10 μm in aerodynamic diameter (PM_10_, daily mean) ranged from 0.16 to 0.70, between CO and black particles with an aerodynamic diameter < 4.5 μm—i.e., black smoke (BS, daily mean)—from 0.67 to 0.82, between CO and sulfur dioxide (daily mean) from 0.35 to 0.82, between CO and NO_2_ (1-hr maximum) from 0.03 to 0.68, and between CO and O_3_ (1-hr maximum) from −0.25 to −0.65. There was substantial variability among cities in the levels of all pollutants and in mean daily temperature.

### Methods

We used a hierarchic modeling approach. First, we fit regression models in each city separately to allow specific control for seasonal effects, weather, and other potential confounders. We used the results of the individual city analysis, in turn, in a second-stage analysis to provide overall estimates and to investigate potential effect modifiers.

#### Individual city analysis

We investigated the CO–mortality associations for each city using Poisson regression models allowing for overdispersion. The city-specific model is of the form:


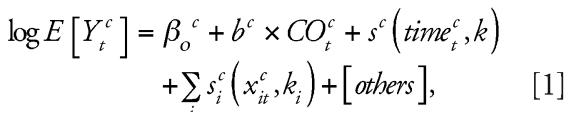


where *E*[*Y_t_^c^*] is the expected value of the Poisson-distributed variable *Y_t_^c^* indicating the daily mortality count on day *t* at city *c* with *Var*(*Y_t_^c^*) = ϕ*E*[*Y_t_^c^*], ϕbeing the overdispersion parameter, *x_it_^c^* is the value of the *x_i_* meteorologic covariate on day *t* at city *c*, *CO_t_^c^* is the air pollution level on day *t* at city *c*. The smooth functions *s* capture the nonlinear relationship between the time-varying covariates and calendar time and daily mortality. We used penalized regression splines as smoothing functions, as implemented by Wood (2000) in R. In this setting, *k* is the number of basis functions. We also included dummy variables for the day of the week effect and holidays.

According to the APHEA-2 methodology (Touloumi et al. 2004), the smooth function of time serves as a proxy for any time-dependent outcome predictors or confounders with long-term trends and seasonal patterns not explicitly included in the model. Hence we removed long-term trends and seasonal patterns from the data to guard against confounding by omitted variables. Weather variables, which are associated with both mortality and CO exposure, were also included. Specifically, we included penalized splines of temperature on the day of death and the day before death in the models. We used natural splines as basis functions for the penalized regression splines. Based on our experiences from previous analyses of the APHEA-2 data, we chose the number of basis functions (*k*) to be 50 for each year of available data for the time variable and 10 for the weather variables, because we considered this number of basis functions sufficient to catch very small fluctuations in the analyzed time series. To control the amount of smoothing separately in each city, we then chose the smoothing parameters that minimized the absolute value of the sum of partial autocorrelation functions (PACFs) of the residuals from lags 3 to 30. To account for nonremovable serial correlation in the residuals, we added autoregressive terms into the model as appropriate (Brumback et al. 2000). In the special case of small cities (and especially in cause-specific mortality with small counts of daily events), we have noticed in previous analyses that the above criterion may lead to undercontrolling for season. In those cases, we allowed more degrees of freedom (df) for time, provided that this imposed only a minor burden on the sum of the residual PACFs. Hence, if such a case occurred, we used at least 1 df/year.

To evaluate how sensitive our results were to the choice of the degree of smoothing, we also applied models using 3, 8, and 12 df/year of available data for the time smooth.

We did not control for influenza epidemics because previously published results (Braga and Zanobetti 2000; Touloumi et al. 2005) have shown that these do not bias the association between air pollution and mortality. It is unclear why the specific time within a winter that an epidemic occurs in a particular city should have much to do with air pollution levels and hence confound the relation under investigation. Nevertheless, because previous studies have focused on particles’ effects to further explore the potential confounding effects of influenza on CO, we repeated the analysis for total mortality adjusting for flu epidemics in the model using 8 df/year for seasonality control. We used the APHEA-2 method for influenza control (Touloumi et al. 2004), including a dummy variable taking the value of one when the 7-day moving average of the respiratory mortality was greater than the 90^th^ percentile of its city-specific distribution.

To investigate potential confounding effects from other pollutants, we also applied two pollutant models. In addition to CO, we alternatively included PM_10_, BS, SO_2_, NO_2_, or O_3_ (1 hr) in our model.

We investigated the exposure–response relationship between CO and mortality using threshold analysis. We selected a grid of threshold values, ranging from 0.0 to 3.0 mg/m^3^ by increments of 0.5—i.e., 0.0, 0.5, 1.0, 1.5, 2.0, 2.5, 3.0 mg/m^3^. For each threshold value *h* we fit the corresponding city-specific model. In the threshold model, we included the pollutant term in the model in the form (pollutant-*h*)^+^, where *x*^+^ = *x* if *x* ≥0 and *x*^+^ = 0 if *x* < 0 and *h* is the threshold value. We then computed the deviance of the fitted model and summed the deviances for that given threshold over all cities. We repeated for all threshold values and finally plotted the sums of the deviance versus the threshold values. Because deviance is a measure of goodness of fit for the model, a possible threshold is the one corresponding to the minimum sum of the city-specific models’ deviance.

#### Second-stage analysis

After we analyzed the average of lags 0 and 1 of CO in each city, we assumed the city-specific effect estimates *b^c^* to be normally distributed around an overall estimate. To see whether some of the variability in the estimates *b^c^* was explained by city characteristics, we estimated fixed-effects pooled regression coefficients by weighted regression of the city-specific estimates *b^c^* on potential effect modifiers (at city level) with weights inversely proportional to the variances of the estimates *b^c^*. If substantial heterogeneity remained across city results beyond the variation associated with the effect modifiers, random-effects regression models were applied. In these models, it was assumed that city-specific estimates *b^c^* were a sample of independent observations from a normal distribution with the same mean and variances equal to the between-cities variance and the squared standard error of *b^c^*. The random variance component was estimated by iteratively reweighted least squares (Berkey et al. 1995).

Potential effect modifiers used in the analysis included variables describing *a*) the air pollution level and mix in each city (i.e., mean levels of CO, PM_10_, BS, NO_2_, O_3_, SO_2_, ratio of PM_10_:NO_2_); *b*) the exposure (number of CO monitors, correlation between monitors’ measurements); *c*) the health status of the population (percentage of cardiorespiratory deaths over total, crude mortality rate, directly standardized mortality rate using the age distribution of the European population as standard, age structure described as percentage > 65, > 75, and < 15 years of age); *d*) the geographic area (northern, western, and central-eastern European cities); and *e*) the climatic conditions (mean temperature and relative humidity levels). Unfortunately, there are very sparse comparable socioeconomic status (SES) indicators across the different countries in Europe available at city level. In particular, the only SES indicator available was the percent of unemployed.

## Results

**Figure 1:**
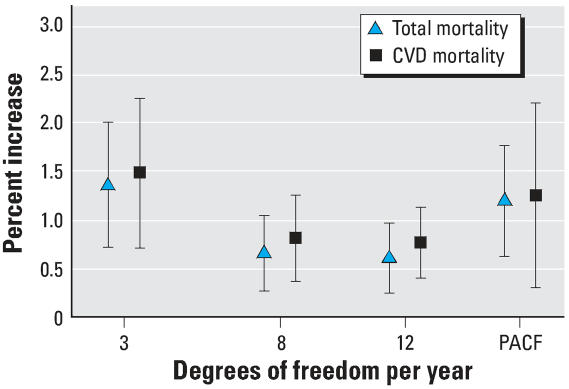
Estimated pooled percent increase in total and cardiovascular mortality and its 95% confidence intervals associated with an increase of 1 mg/m^3^ in the level of CO (average of lags 0–1), using different methods for seasonality control—i.e., with a fixed number of annual degrees of freedom and with variable numbers of annual degrees of freedom according to the PACF.

Figure 1 shows the pooled percentage increase in the daily number of deaths from all causes and from cardiovascular causes associated with 1-mg/m^3^ increase in CO levels, for different degrees of seasonality control. Because there was significant heterogeneity in the single-city results, pooled estimates using random-effects models are shown. Using the PACF criterion, a 1-mg/m^3^ increase in the 2-day mean of CO levels, was associated with a 1.20% [95% confidence interval (CI), 0.63–1.77%] increase in total deaths and a 1.25% (95% CI, 0.30–2.21%) increase in cardiovascular deaths. The PACF criterion used on average 5 df/year for seasonality control when analyzing total mortality and 4 df/year for the analysis of cardiovascular mortality. When we adjusted for seasonality using 3 df/year (i.e., fewer degrees of freedom compared with the PACF), the estimated effects increased by < 20% compared with the model using the PACF criterion. When we adjusted for seasonality using 8 and 12 df/year (i.e., more degrees compared with the PACF), the estimated effects decreased by approximately 40%. As expected, increasing the degrees of freedom for seasonality control leads to a decrease of the magnitude of the effect and consequently of the amount of the observed heterogeneity. When we analyzed total mortality using 8 df/year for seasonality control and also adjusting for influenza epidemics, the pooled effect was decreased only by 7% [associated estimate 0.61%; 95% CI, 0.22–1.02%)], supporting the hypothesis of absence of confounding by influenza epidemics.

**Figure 2:**
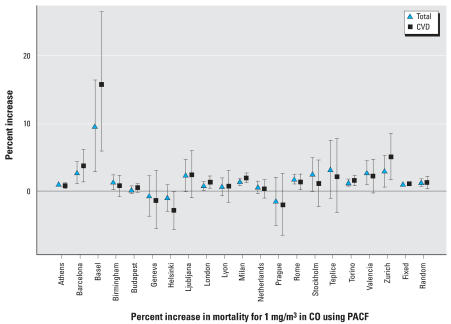
Percent increase in the daily number of deaths from all and cardiovascular causes and their 95% CIs associated with an increase of 1 mg/m^3^ in the level of CO in each city and overall cities, as estimated using the PACF criterion.

Figure 2 presents the percentage increase in the daily number of deaths from all causes and cardiovascular causes and their 95% CI associated with an increase of 1 mg/m^3^ in the 2-day mean of CO levels in each city and in all cities, as estimated from the models using the PACF criterion for seasonality control. The statistically significant estimated increases in total mortality per 1-mg/m^3^ increase in CO ranged from 0.98% in Athens to 9.39% in Basel, whereas the corresponding increases in cardiovascular mortality ranged from 0.76% in Athens to 15.69% in Basel. Because Basel is a small city it has a small weight on the pooled estimates; hence, although it is an outlier it does not greatly affect the pooled estimates. So when we excluded Basel from the pooled estimates, a 1-mg/m^3^ increase in the 2-day mean of CO levels, was associated with 1.11% increase (95% CI, 0.69–1.53%) in total mortality and 1.13% increase (95% CI, 0.50–1.77%) in cardiovascular mortality.

**Figure 3:**
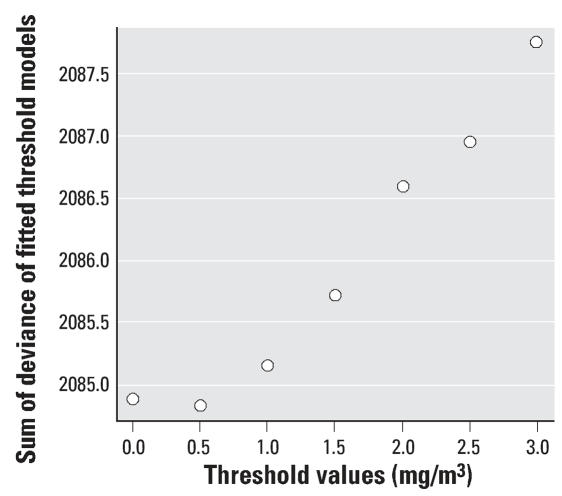
Total deviance of different fixed threshold models for the relationship between CO and total mortality.

Figure 3 presents the sum over all cities of the city-specific deviances for different threshold models versus the different threshold values explored in the analysis of CO and total mortality. The overall deviance was minimized for a potential threshold of 0.5 mg/m^3^; however, the comparison between the deviance of that threshold model and the linear no-threshold model yields a *p*-value > 0.9, suggesting weak evidence for a threshold.

Table 2 presents results from two-pollutant models, adjusting in turn for the confounding effects of BS, PM_10_, SO_2_, O_3_, and NO_2_. CO associations with both total and cardiovascular mortality were confounded by BS and NO_2_ under both methods for seasonality control, but remained marginally significant. When adjusting for NO_2_, the estimated increase in total mortality associated with an increase of 1 mg/m^3^ in the 2-day mean of CO-levels was reduced by 27% using the PACF method and by 55% using 8 df/year, and the corresponding increase in cardiovascular mortality was reduced by 33% under PACF and 47% under 8 df/year. When adjusting for BS, the estimated increase in total mortality associated with an increase of 1 mg/m^3^ in the 2-day mean of CO was reduced by 36% under PACF and 32% under 8 df/year, and the corresponding increase in cardiovascular mortality was reduced by 34% under PACF and 40% under 8 df/year. The highest effects estimate for CO was found when ozone was included in the two-pollutant model.

Table 3 shows the resulting estimated CO effect (that is, the increase in mortality and its 95% CI per 1-mg/m^3^ increase in the 2-days mean of CO) for a city characterized by a value in the effect modifier equal to the 25^th^ (low) and 75^th^ (high) percentile of the distribution of this particular effect modifier. Among the potential effect modifiers, we present only those that explained > 10% of the observed heterogeneity in the estimates obtained both by the PACF criterion and using 8 df/year for seasonality control.

When we investigated the source of heterogeneity in the association between CO and total mortality, the most important effect modifiers were the geographic area (defined as western, southern, and central-eastern European cities) and the age-standardized mortality rate. More specifically, the effect of CO on total and cardiovascular mortality was highest in western cities, followed closely by the effect in southern European cities, whereas there was only a small and nonsignificant effect in eastern cities. Higher effects of CO on mortality are observed in cities with lower morality rates. In addition, higher effects of CO on total mortality are observed in cities with smaller number of CO monitors and higher particle levels, whereas the effect on cardiovascular mortality is higher in cities with lower O_3_ levels and higher proportion of elderly.

## Discussion

We used the most extensive European database available today to investigate the effects of CO on mortality from all causes and cardiovascular causes. We found significant associations of CO with total and cardiovascular mortality. The slightly stronger effect of CO on cardiovascular mortality is expected, taking into consideration its biological mechanism and the fact that deaths from cardiovascular causes account for about 45% of the total number of deaths in the analyzed cities. The results of our threshold analysis support the hypothesis of a linear exposure–response relationship between CO and mortality. The present findings complement those previously reported from APHEA-1, which was the first part of the APHEA project and included a smaller number of cites (i.e., for CO only 1, compared with 19 cites included in the APHEA-2 analysis). As part of that analysis, Touloumi et al. (1996) reported significant positive associations between CO and daily total mortality in Athens, Greece. In that first part of the APHEA analysis, an increase per 1 mg/m^3^ in CO was associated with 1% (95% CI, 0.5–1.5%) increase in total mortality, which is in agreement with the 1% (95% CI, 0.7–1.3%) increase in Athens reported in our results. Statistically significant associations between CO and total mortality have previously been reported in Los Angeles, California (Kinney and Ozkaynak 1991; Shumway et al. 1988), the Netherlands (Fischer et al. 2003), Russia (Katsnel’son et al. 2000), and Canada (Burnett et al. 1998a, 1998b). In particular, Burnett et al. (1998b) reported that the effects of the complex air pollution mixture could be explained almost completely by the levels of CO and total suspended particulates.

Because a single pollutant could act as a marker of a pollution mixture, the independence of the adverse effect of CO on mortality from those of other pollutants is unclear. Hence, CO could be a marker of other pollutants generated by vehicle exhausts such as particles. We have tried to separate the independent effects of pollutants using two-pollutant models. There was evidence that CO effects on total and cardiovascular mortality were confounded by BS and NO_2_. Because the pollutant-adjusted effects of CO remained at least marginally statistically significant, there appears to be a persistent effect of CO additional to that of the other traffic-related pollutants. The reduction of the CO effects was in most cases higher when we allowed for 8 df/year for seasonality control, but it is expected that the inclusion of additional pollutants will have a greater effect on the smaller effect estimates produced from increasing degrees of freedom for seasonality. Because CO had the highest correlations with BS, the BS-adjusted estimates are limited by the collinearity among pollutants generated by the same source. On the other hand, the BS effect on mortality (obtained under the same methodology) is reduced by the same (in the case of total mortality) or even greater (in the case of cardiovascular mortality) amount after the inclusion of CO, indicating the collinearity of these pollutants but also the persistence of the CO effect on cardiovascular mortality. To a lesser degree, CO seems to have a confounding effect on the NO_2_ effects on mortality, especially under the PACF method. The NO_2_-adjusted effect estimates of CO may reflect, to a larger extent, the effects from sources other than traffic. Further study focusing on exposure to mixtures including NO_2_, BS, and CO is needed to further our understanding of the etiologic mechanism through which the pollutants affect mortality. The highest effects estimate for CO was found when O_3_ was included in the two-pollutant model, due to the O_3_ effect on mortality together with their negative correlation, which is especially high in winter. This relation between two harmful types of air pollutants is also seen in the estimation of O_3_ effects adjusting for CO (Gryparis et al. 2004).

We investigated several environmental and social factors that might explain variation between cities in the effect estimates. The effect of CO on total and cardiovascular mortality was observed in western and southern European cities, whereas there was essentially no CO effect in eastern cities. This geographic difference was also observed in the effects of the other pollutants analyzed within the APHEA project (Gryparis et al. 2004; Katsouyanni et al. 2001; Samoli et al. 2006). Hence any variables, such as the mortality rate, that strongly differ between eastern and other regions are likely to appear as significant explanatory factors of the heterogeneity in the effect estimates. Alternatively, if the effect modifiers identified are indeed true modifiers, their distribution across Europe may result in these marked geographic differences. In agreement with this possibility is the result that cities with larger number of CO monitoring stations have lower effect of CO on total mortality, because three of the four eastern cities in our analysis have a large number of CO monitoring stations (above the 75^th^ percentile of this effect modifier’s distribution). Furthermore, in cities with higher PM_10_ levels, the effect of CO on total mortality is higher because traffic is a predominant source of particles in European cities with high PM concentrations. For cardiovascular mortality, the effect of CO is higher in cities with lower standardized mortality rate and higher proportion of elderly. In fact, in the APHEA cities, a large mortality rate was related to a smaller proportion of elderly persons (Spearman *r* = −0.33). An explanation may be that a low standardized mortality rate and a high percentage of elderly implies a larger proportion of individuals belonging to vulnerable groups who are more susceptible to pollution effects or, complimentarily, that in a population with higher mortality there are more competing risks. Finally, the result that in cities with higher O_3_ levels the CO effect on cardiovascular mortality is lower may reflect a stronger effect of photochemical air pollution on mortality in the cities where such a pollution mix is dominant. The situation is also complicated by the fact that CO is involved in atmospheric photochemistry.

In conclusion, we found adverse effects of CO on mortality from all causes and cardiovascular causes using the most extensive European database. Although there was indication of confounding by other vehicle-derived pollutants, the unexplained short-term effect of CO on mortality remained at least marginally statistically significant. The heterogeneity found in the effect estimates among cities may be explained partly by factors characterizing the air pollution mix and the health of the population.

## Figures and Tables

**Table 1:** City descriptive data on the study period, population, exposure (CO), outcome (daily number of deaths), and potential effect modifiers.

Location	Study period (month/year)	Population (×1,000)	Total no. of deaths	CVD	CO (mg/m^3^) [mean (10^th^–90^th^)]^a^	No. of CO monitors	PM (mean)	O_3_ (mean)	SMR	% of population> 75 years of age	Area
Athens	1/92–12/96	3,073	73	36	6.1 (3.5–9.2)	2	42.7	87.1	784	5.3	South
Barcelona	1/91–12/96	1,644	40	16	0.9 (0.4–1.7)	3	63.5	71.9	740	7.3	South
Basel	1/90–12/95	360	9	4	0.6 (0.4–1.1)	1	31.2	64.0	678	7.6	Northwest
Birmingham	1/92–12/96	2,300	61	28	1.0 (0.5–1.6)	1	24.5	55.7	895	6.5	Northwest
Budapest	1/92–12/95	1,931	80	40	5.1 (3.3–7.4)	8	41.0	83.8	1,136	6.6	Central-eastern
Geneva	1/90–12/95	317	6	2	1.5 (0.8–2.6)	4	39.7	67.3	608	6.4	Northwest
Helsinki	1/93–12/96	828	18	9	1.2 (0.7–1.9)	2	27.7	58.0	915	5.0	Northwest
Ljubljana	1/92–12/96	322	7	3	1.6 (0.6–3.0)	1	—	80.0	823	4.5	Central-eastern
London	1/92–12/96	6,905	169	71	1.4 (0.7–2.2)	3	28.8	44.9	851	6.5	Northwest
Lyon	1/93–12/97	416	9	3	3.8 (2.0–6.0)	4	41.8	66.6	579	8.5	Northwest
Milano	1/90–12/96	1,343	29	11	5.4 (2.9–8.7)	3	53.0	52.7	623	9.6	Northwest
Netherlands	1/90–9/95	15,400	342	143	0.6 (0.4–1.2)	5	40.1	69.8	757	5.5	Northwest
Prague	2/92–12/96	1,213	38	22	0.9 (0.5–1.5)	4	76.2	85.3	984	5.7	Central-eastern
Rome	1/92–12/96	2,775	56	23	4.1 (2.5–5.9)	5	58.7	47.5	585	6.2	South
Stockholm	1/90–12/96	1,126	30	15	0.8 (0.5–1.2)	2	15.6	65.0	666	11.0	Northwest
Teplice	1/90–12/97	625	18	8	0.7 (0.3–1.2)	5	47.9	55.5	1,173	3.5	Central-eastern
Torino	1/90–12/96	926	21	9	5.5 (2.8–9.1)	2	74.5	88.6	724	7.8	Northwest
Valencia	1/94–12/96	753	16	6	4.1 (2.4–5.9)	4	—	59.1	820	5.5	South
Zurich	1/90–12/95	540	13	6	1.2 (0.7–2.0)	3	31.3	66.8	666	7.2	Northwest

Abbreviations: —, no data; SMR, standardized mortality rate.

a Percentiles.

**Table 2:** Pooled estimates (95% CIs) for the percent increase in mortality associated with an increase of 1 mg/m^3^ in CO (average of lags 0 and 1), adjusting alternatively for the other pollutants.

	Total mortality				CVD mortality			
	8 df/year		PACF		8 df/year		PACF	
Other pollutant	Fixed effects	Random effects	Fixed effects	Random effects	Fixed effects	Random effects	Fixed effects	Random effects
None	0.59 (0.41 to 0.78)	0.66 (0.27 to 1.05)	1.00 (0.83 to 1.18)	1.20 (0.63 to 1.77)	0.80 (0.53 to 1.07)	0.81 (0.36 to 1.26)	1.06 (0.80 to 1.32)	1.25 (0.30 to 2.21)
BS	0.35 (−0.03 to 0.72)	0.45 (−0.01 to 0.92)	0.67 (0.30 to 1.04)	0.77 (0.28 to 1.26)	0.49 (−0.04 to 1.02)	0.49 (−0.04 to 1.02)	0.83 (0.31 to 1.35)	0.83 (0.31 to 1.35)
PM_10_	0.48 (0.24 to 0.72)	0.58 (0.12 to 1.04)	0.78 (0.55 to 1.00)	1.09 (0.36 to 1.83)	0.73 (0.39 to 1.07)	0.73 (0.39 to 1.07)	0.95 (0.62 to 1.27)	1.13 (0.60 to 1.67)
SO_2_	0.44 (0.21 to 0.67)	0.46 (0.07 to 0.85)	0.68 (0.47 to 0.90)	0.75 (0.26 to 1.26)	0.72 (0.39 to 1.04)	0.68 (−0.03 to 1.40)	0.91 (0.59 to 1.22)	0.86 (0.06 to 1.66)
O_3_ (1 hr)	0.66 (0.46 to 0.86)	0.76 (0.45 to 1.06)	1.12 (0.93 to 1.31)	1.37 (0.81 to 1.95)	0.91 (0.62 to 1.20)	1.02 (0.58 to 1.46)	1.28 (1.01 to 1.56)	1.62 (0.72 to 2.52)
NO_2_ (1 hr)	0.27 (0.03 to 0.51)	0.30 (−0.11 to 0.71)	0.72 (0.50 to 0.95)	0.88 (0.22 to 1.55)	0.44 (0.10 to 0.79)	0.43 (−0.06 to 0.93)	0.68 (0.35 to 1.00)	0.84 (−0.03 to 1.71)

**Table 3:** Results of second-stage regression models, investigating the role of potential modifiers^a^ of the estimated effects^b^ of CO on mortality.

	Total mortality				CVD mortality			
	8 df/year		PACF		8 df/year		PACF	
Effect modifier^c^	25^thd^	75^thd^	25^thd^	75^thd^	25^thd^	75^thd^	25^thd^	75^thd^
No. of CO monitors	0.71 (0.48 to 0.94)	0.54 (0.34 to 0.74)	1.18 (0.96 to 1.39)	0.92 (0.73 to 1.11)				
Mean PM_10_ levels	0.37 (0.08 to 0.66)	0.49 (0.28 to 0.69)	0.74 (0.46 to 1.02)	1.07 (0.87 to 1.27)				
Mean O_3_					1.04 (0.67 to 1.41)	0.82 (0.55 to 1.10)	1.32 (0.96 to 1.68)	1.09 (0.83 to 1.35)
SMR	0.79 (0.55 to 1.03)	0.44 (0.22 to 0.66)	1.29 (1.06 to 1.52)	0.77 (0.56 to 0.98)	1.06 (0.71 to 1.42)	0.61 (0.30 to 0.93)	1.40 (1.06 to 1.75)	0.85 (0.55 to 1.14)
Population > 75 years of age (%)					0.58 (0.25 to 0.92)	0.94 (0.64 to 1.24)	0.74 (0.41 to 1.06)	1.25 (0.96 to 1.54)
Geographic region								
Western cities	0.75 (0.47 to 1.03)		1.15 (0.90 to 1.40)		1.06 (0.67 to 1.46)		1.38 (1.00 to 1.76)	
Southern cities	0.61 (0.32 to 0.91)		1.08 (0.79 to 1.38)		0.70 (0.26 to 1.14)		0.90 (0.47 to 1.33)	
Eastern cities	0.03 (−0.47 to 0.53)		0.27 (−0.20 to 0.74)		0.21 (−0.48 to 0.90)		0.48 (−0.14 to 1.11)	

SMR, standardized mortality rate.

a Variables characterizing each city; only statistically significant effect modifiers under both methods for seasonality control, reducing the heterogeneity by > 10% are presented.

b Effect estimates used from first-stage models are based on the PACF criterion and on 8 df/year for seasonality control.

c The effect modifiers were included alternatively in the model.

d Percent increase in mortality per 1-mg/m^3^ increase in the 2-day mean CO levels, estimated using fixed-effects model, for a city with levels of the corresponding effect modifier equal to the 25th and the 75th percentiles of its distribution.
